# Dynamics of Multicomponent Vesicles in Narrow Channel

**DOI:** 10.3390/membranes16100312

**Published:** 2026-09-24

**Authors:** Wang Xiao, Pingqia Wang, Kai Liu

**Affiliations:** 1 School of Mathematics and Statistics, Changsha University of Science and Technology, Changsha 410114, China; 2 Faculty of Arts and Sciences, Beijing Normal University, Zhuhai 519087, China

**Keywords:** multicomponent vesicle, lipid phases, Poiseuille flow, boundary integral method

## Abstract

We investigate the dynamics of two-dimensional multicomponent vesicles in confined Poiseuille flow, focusing on the interplay among membrane composition, mechanical heterogeneity, and hydrodynamic confinement. The membrane composition is described by a phase-field model, while the local bending rigidity depends on the phase composition, thereby coupling phase separation to membrane deformation. Local membrane inextensibility is imposed through a Lagrange multiplier, and the coupled vesicle-fluid system is solved using a boundary integral method for matched interior and exterior viscosities. At a fixed fivefold bending-rigidity contrast, we examine the effects of the average phase composition, channel width, and initial vesicle orientation. In contrast to homogeneous vesicles, which generally relax toward symmetric bullet-like configurations near the channel centerline, multicomponent vesicles exhibit pronounced symmetry breaking and a variety of dynamical states. These include asymmetric steady shapes accompanied by membrane tank-treading, as well as confinement-dependent breathing and wagging motions. Mechanical heterogeneity also promotes localized high-curvature regions during transient deformation, with softer membrane domains preferentially occupying regions of larger curvature. These results demonstrate that composition-dependent bending rigidity can qualitatively alter vesicle dynamics in pressure-driven confined flows and highlight the importance of membrane heterogeneity in the transport and deformation of vesicle-like cells in narrow channels.

## 1. Introduction

Multicomponent vesicle flow in confined microchannels is a fundamental moving-boundary problem in mathematical biology, with potential applications to understanding microcirculatory dysfunction in sickle cell disease (SCD). SCD is a hereditary hematological disorder driven by the abnormal polymerization of intracellular hemoglobin S. This process alters red-cell hydration and membrane mechanics and produces marked heterogeneity in cell density, morphology, and deformability [1,2]. These alterations can lead to abnormal deformation and motion of RBCs in confined capillaries (with diameters ranging from 5 to 15 μm), increasing flow resistance and the risk of vascular adhesion, thereby contributing to capillary occlusion and distal tissue ischemia [3,4,5]. It is therefore useful to develop a mathematical model that accounts for membrane mechanical heterogeneity when describing vesicle dynamics in confined Poiseuille flow.

The deformation of RBCs in microvessels is governed by an interplay among their surface area, volume, membrane mechanical properties, and hydrodynamic interactions with the surrounding flow [6,7]. Microvascular resistance plays a major role in determining peripheral blood flow [8]. This resistance depends on both the architecture of the microvascular network and the rheological properties of blood, which are strongly influenced by the shape and deformability of RBCs [6,8]. The dynamics of deformable vesicles immersed in a viscous fluid confined within a narrow channel constitutes a canonical moving-boundary problem in continuum mechanics and mathematical biology. The problem is characterized by two core nonlinear couplings: the fluid–structure interaction between the vesicle membrane and the surrounding flow [9,10,11], and the interphase mechanical interaction between different lipid phases on the vesicle membrane [12,13,14,15,16]. For confined Poiseuille flow with a low Reynolds number (Re≪1), fluid inertia is negligible, and the flow field is driven by the pressure gradient and the velocity of the moving vesicle membrane. The mathematical difficulty lies in the strong nonlinearity of the free-boundary problem: membrane-shape evolution changes the boundary conditions of the flow field, which in turn affects subsequent membrane deformation [12,16,17]. Moreover, membrane inextensibility and phase-separation-induced mechanical heterogeneity further complicate the stable numerical solution of the problem [12,18].

Boundary integral and boundary element methods have been extensively used to simulate their morphology and migration in Poiseuille flow, revealing a rich variety of steady shapes—including symmetric bullet-like, parachute-like, slipper-like, and snaking configurations—whose transitions are governed by the reduced volume, capillary number, and confinement ratio [7,19]. In simple shear flow, the transition from tank-treading to tumbling (or trembling) motion has been systematically investigated, with the critical conditions shown to depend on the viscosity contrast between the interior and exterior fluids as well as on the excess area [20]. In parallel, multicomponent vesicle models based on the phase-field method have been developed to account for membrane heterogeneity [12,21,22]. Thermodynamically consistent phase-field formulations were designed to capture spinodal decomposition, coarsening, budding, and fission—morphological processes driven by differences in bending rigidity and spontaneous curvature among coexisting lipid phases [12,21]. Complementarily, sharp-interface boundary integral approaches for multicomponent vesicles in Stokes flow demonstrated that an initially elliptical multicomponent vesicle tank-treads and attains a steady shape and surface phase distribution under applied shear, while a sufficiently elongated vesicle tumbles with complex morphological evolution in regions where the bending rigidity and spontaneous curvature are small [22]. Subsequent phase-field studies further examined how the phase fraction and the mechanical contrast between phases influence morphological evolution [23].

Despite these advances, the dynamics of multicomponent vesicles in straight, pressure-driven channels remain incompletely characterized. This gap motivates the present work. We establish a fully coupled mathematical model for multicomponent vesicles in confined Poiseuille flow and use a stable numerical scheme. Following previous works [12,18,24], the phase-field–boundary integral formulation combines the Helfrich bending energy of the heterogeneous membrane with the Stokes equations. A numerical scheme combining the Fourier pseudospectral method and small-scale decomposition is used. At a fixed fivefold bending-rigidity contrast, we analyze the effects of average phase concentration, channel width, and initial vesicle orientation. To simplify the model, we assume that the fluids inside and outside the vesicle have the same viscosity and that the vesicle membrane initially contains a nearly uniform mixture of the two phases.

Using capillary blood flow as motivation, we isolate the effects of bending rigidity and membrane heterogeneity within an idealized, locally inextensible vesicle model. Our numerical results reveal that an inhomogeneous bending modulus leads to rich dynamics in Poiseuille flow beyond the familiar regimes of a homogeneous vesicle.

The paper is organized as follows. In Section 2, we introduce the physical model and briefly describe the boundary integral numerical method. In Section 3, we present and discuss simulations of vesicle dynamics in a narrow channel. Section 4 discusses limitations and directions for future work.

## 2. Modeling and Methods

For simplicity, we consider a closed multicomponent vesicle whose interior and exterior are filled with fluids of the same viscosity μ. The vesicle is located in a narrow channel driven by a pressure difference between the inlet and outlet.

### 2.1. Modeling

#### 2.1.1. Modeling of the Vesicle

We first introduce the material field. We consider a vesicle membrane containing two coexisting membrane domains. The phase variable ψ(s,t) represents the local membrane composition; the two limiting phases may be viewed as domains with different lipid compositions and, at the model level, different bending rigidities. Here *s* is the arclength parameterizing the moving interface S; the concentration of the other phase is 1−ψ(s,t). The line energy associated with the membrane phases is

(1) $$E_{\psi }=\frac{a}{\epsilon }\int_{S}\left(g\left(\psi \right)+\frac{\epsilon^{2}}{2}\psi_{s}^{2}\right)\;dS,$$

The function $g\left(\psi \right)=\frac{1}{4}\psi^{2}{\left(1-\psi \right)}^{2}$ is a double-well potential with minima at the two pure phases, ψ=0 and ψ=1. The parameters *a* and ϵ set the magnitude of the line energy and the diffuse-interface thickness, respectively.

The total mass reads

(2) $$M_{\psi }\left(t\right)=\int_{S}\psi \left(s,t\right)\;ds=M_{\psi }\left(0\right),$$

where the surface density of each phase is taken to be unity.

We assume that no chemical reactions occur and that both phases are confined to the interface S, so the amount of each phase is conserved. In Lagrangian coordinates on a locally inextensible membrane, the surface Cahn–Hilliard equation reduces to

(3) $$\psi_{t}=J_{s},$$

where J is the surface flux defined below in Equation (9).

The total energy of the system

(4) $$\begin{array}{ccc} E & = & E_{b}+E_{p}+E_{\psi }\;\mathrm{with} \end{array}$$

(5) $$\begin{array}{ccc} E_{b} & = & \frac{1}{2}\int_{S}B\left(\psi \right)H^{2}\;dS,\; \end{array}$$

(6) $$\begin{array}{ccc} E_{p} & = & \int_{S}\Lambda \;dS,\; \end{array}$$

where $E_{b}$ is the bending energy, B(ψ) is the composition-dependent bending rigidity, and *H* is the curvature. We use the linear interpolation $B\left(\psi \right)=\left(1-\psi \right)B_{1}+\psi B_{2}$. The reference value $B_{1}=3.0\times 10^{-19}\;J$ sets an RBC-scale bending resistance [25]. We assign $B_{1}$ to the red phase and choose $B_{2}=5B_{1}$ for the stiffer blue phase as an illustrative mechanical contrast. Note that the cited RBC bending measurement is not evidence for a fivefold local contrast within a sickle-cell membrane. $E_{p}$ is the energy due to membrane tension Λ, which can be viewed as a Lagrange multiplier that enforces the local inextensibility constraint.

The bending energy assumes zero spontaneous curvature. The linear law follows the constitutive choice in [22]; it is not an experimentally calibrated composition law. Linear interpolation avoids additional constitutive parameters, and its constant derivative $B_{\psi }=B_{2}-B_{1}$ simplifies the coupling between bending and phase evolution. Most of the membrane lies near the pure-phase endpoints, with relatively narrow transition regions, which motivates this approximation for the qualitative model. Nonlinear laws with steeper interfacial variations could increase numerical stiffness.

The membrane force density is obtained by varying the total membrane energy with respect to the interface position,

(7) $$f=-\frac{\delta E}{\delta S},$$

which gives

(8) $$\begin{array}{ccc} f & = & \left({\left(BH\right)}_{ss}+\frac{B}{2}H^{3}-\frac{a}{\epsilon }\left(g-\frac{\epsilon^{2}}{2}\psi_{s}^{2}\right)H-\Lambda H\right)n\\ & & -\left(\left(\frac{a}{\epsilon }\left(g^{\prime }-\epsilon^{2}\psi_{ss}\right)+\frac{B^{\prime }}{2}H^{2}\right)\psi_{s}-\Lambda_{s}\right)s, \end{array}$$

where ${\left(\cdot \right)}_{s}$ and ${\left(\cdot \right)}^{\prime }$ indicate differentiation with respect to the arclength and ψ, and n, s are the outward normal and tangential direction of the interface S respectively.

The surface flux is then defined as

(9) $$J=\nu \nabla_{s}w,$$

where ν>0 is the phase mobility and sets the rate of diffusive redistribution in response to the full chemical-potential gradient. The chemical potential *w* is defined by

(10) $$w=\frac{\delta E}{\delta \psi }=\frac{1}{2}B_{\psi }H^{2}+\frac{a}{\epsilon }\left(g_{\psi }-\epsilon^{2}\psi_{ss}\right).$$

Phase redistribution is driven by the gradient of the complete chemical potential in Equation (10). Its three contributions have different roles: the double-well potential favors demixing, the gradient term penalizes sharp phase boundaries, and the bending term couples composition to curvature. Since $B_{\psi }=B_{2}-B_{1}>0$, placing the stiffer phase at large |H| raises the bending-energy cost; all else being equal, the softer phase is therefore favored in more strongly curved regions. Diffusion competes with transport by the moving membrane and with the changing curvature generated by the flow. In particular, even if $B_{1}=B_{2}$, the mixing and interfacial terms can still drive phase separation. Bending contrast is not the sole driving force. Phase separation can also occur on a circle with uniform curvature. Curvature influences the spatial arrangement of the separated phases through the chemical potential; the resulting redistribution of ψ then changes the local modulus through B(ψ). Curvature does not directly change *B* at fixed composition. The evolution equation of the phase distribution reads as

(11) $$\psi_{t}=\nu \left(\frac{a}{\epsilon }{\left(g^{\prime }-\epsilon^{2}\psi_{ss}\right)}_{ss}+B^{\prime }\left(H_{s}^{2}+{HH}_{ss}\right)\right).$$

The most direct physical analog is a multicomponent fluid bilayer with coexisting domains, for which correlations between composition and curvature have been observed experimentally [26]. In the present model, ψ is a membrane composition variable, not the intracellular polymerized-HbS fraction. No oxygen-dependent reaction or HbS-fiber stress is included. RBC measurements distinguish bending and shear moduli from cytoplasmic viscosity and show that the mechanical changes depend on the cell population and measurement conditions [27]. These measurements do not calibrate a local ratio $B_{2}/B_{1}=5$. Thus, the chosen ratio is a model parameter used to examine finite bending heterogeneity, not a universal normal-to-sickle stiffness ratio. Local inextensibility constrains membrane stretching but does not supply the spectrin network’s in-plane shear elasticity. A quantitatively predictive RBC model would additionally require cytoskeletal mechanics, intracellular rheology and polymerization, and three-dimensional geometry.

#### 2.1.2. Flow Field

We use the same viscosity for the interior and exterior fluids for simplicity. The Reynolds number of Poiseuille flow in a narrow channel is small, $Re∼8.3\times 10^{-3}$, for a channel width W=10μm [28], viscosity μ=1.2 mPa·s [28], characteristic velocity (the maximum cell-free channel velocity) $u_{\max}∼10^{-3}$ m/s [29], and mass density $\rho =10^{3}$ kg/m^3^.

We consider a unit cell $\Omega_{0}$ confined by one period of the upper and lower walls, denoted by *U* and *D*, respectively. The full periodic channel is $\Omega ={⋃}_{n\in Z}\left(\Omega_{0}+nd\right)$, where d=(L,0) is the lattice vector and *L* is the streamwise period. Let $u\left(x\right)=(u_{1}\left(x\right),u_{2}\left(x\right))$ be the velocity field and p(x) the pressure for $x=\left(x_{1},x_{2}\right)\in R^{2}$. For a positive pressure drop ΔP in the positive $x_{1}$ direction, the pair (u,p) satisfies

(12) $$\begin{array}{ccc} -\mu \Delta u+\nabla p & = & 0, \end{array}$$

(13) $$\begin{array}{ccc} \;\nabla \cdot u & = & 0, \end{array}$$

(14) $$\begin{array}{ccc} \;u & = & 0\;\mathrm{on}\;U\;\mathrm{and}\;D, \end{array}$$

(15) $$\begin{array}{ccc} \;u(x+d)-u(x) & = & 0,\;x\in \Omega , \end{array}$$

(16) $$\begin{array}{ccc} \;p(x+d)-p(x) & = & -\Delta P,\;x\in \Omega . \end{array}$$

Across the interface S, the velocity is continuous:

(17) $${\left[\;\left[u\right]\;\right]}_{S}\;:=\;u{{|}_{S,\;\mathrm{int}}-u|}_{S,\;\mathrm{ext}}=0.$$

The hydrodynamic stress sustains a jump given by the Laplace-Young condition:

(18) $${\left[\;\left[Tn\right]\;\right]}_{S}=f,$$

where T is the fluid stress.

Finally, imposing a no-slip boundary condition at the vesicle boundary yields the following kinematic condition:

(19) $$\dot{x}=u\;\mathrm{on}\;S,$$

where $\dot{x}$ is the velocity of a material point on the vesicle membrane. The local inextensibility reads

(20) $$\nabla_{s}\cdot u=0.$$

The capillary number, which measures the ratio of viscous forcing to bending resistance, is defined as $Ca=\mu \dot{\gamma }R^{3}/B_{1}$, where $\dot{\gamma }=2U_{\max}/W$ and R=l/(2π) is the effective vesicle radius based on its perimeter *l*.

### 2.2. Numerical Scheme

In summary, the model requires us to solve the Stokes Equations (12)–(16), subject to the local inextensibility condition (20) and boundary conditions (17) and (18) with the stress jump f given by the constitutive Equation (7), together with a high-order Cahn-Hilliard type Equation (3) with the surface flux given by the constitutive Equation (9). Our numerical scheme is based on the work of [12,24]. We provide a brief summary of the method here.

#### 2.2.1. Boundary Integral Equations for the Flow Field

A boundary integral equation (BIE) is used to solve the fluid problem [24]. For two-dimensional Stokes flow in a periodic channel of period *d*, the periodic boundary integral equation (BIE) can be formulated in terms of single-layer potentials when the viscosity ratio across particle interfaces equals unity. The classical periodic single-layer representation constructs the periodic kernel by summing free-space Stokeslets over all periodic images. For a unit cell Ω bounded by upper and lower walls Γ=U∪D, the velocity field induced by a surface force density τ on the walls reads

(21) $$u\left(x\right)=S_{\Gamma }^{P}\tau =\int_{\Gamma }S^{P}\left(x,y\right)\tau \left(y\right)ds_{y},$$

where the periodic single-layer kernel is defined as $S^{P}\left(x,y\right)=\sum_{n\in Z}S\left(x,y+nd\right)$, with S(·,·) the free-space Stokes single-layer kernel (stokeslet) and d the lattice vector of the periodic array. Enforcing the no-slip Dirichlet condition ${\left.u\right|}_{\Gamma }=v$ on the channel walls yields the first-kind periodic BIE

(22) $$S_{\Gamma ,\Gamma }^{P}\tau =v.$$

Instead of evaluating the infinite lattice sum directly, the present method decomposes the periodic flow into near-field image contributions and a smooth far-field correction, retaining only free-space single-layer kernels to reuse existing fast multipole implementations and high-order singular quadratures. The near-field component sums free-space single-layer potentials over the central unit cell and its immediate left and right neighboring images (|n|≤1), while the contribution from all remaining distant periodic images is approximated by a small set of auxiliary Stokeslets (proxy sources) placed on a circular contour enclosing the unit cell. The velocity representation inside the unit cell therefore takes the form

(23) $$u\left(x\right)=S_{\Gamma }^{\mathrm{near}\;}\tau +\sum_{m=1}^{M}c_{m}\phi_{m}\left(x\right),$$

where $S_{\Gamma }^{\mathrm{near}\;}$ denotes the sum of free-space single-layer operators over the three nearest periodic copies, $\phi_{m}\left(x\right)=S\left(x,y_{m}\right)$ are the single-layer velocity fields generated by the *M* proxy sources at positions $y_{m}$, and $c_{m}\in R^{2}$ are the unknown proxy strength coefficients.

Periodicity and wall boundary conditions are imposed simultaneously by assembling an extended linear system (ELS). On the upper and lower walls, the no-slip Dirichlet condition gives the wall velocity block

(24) Aτ+Bc=v,

where $A=S_{\Gamma ,\Gamma }^{\mathrm{near}}$ maps wall force densities to wall velocities, *B* maps proxy source strengths to wall velocities, and v is the prescribed Dirichlet velocity data on the walls. On the left and right boundaries $\Gamma_{L}$ and $\Gamma_{R}=\Gamma_{L}+d$ of the unit cell, periodicity requires continuity of velocity and a prescribed traction jump corresponding to the applied pressure drop across one period. This gives the periodic discrepancy block

(25) Cτ+Qc=g,

where *C* and *Q* map wall densities and proxy strengths, respectively, to the velocity and traction mismatches between $\Gamma_{L}$ and $\Gamma_{R}$, and g encodes the imposed pressure drop ΔP as a traction jump across the period.

For vesicle suspensions with unit interior-exterior viscosity ratio, the disturbance flow induced by vesicle membrane forces is also represented purely via free-space single-layer potentials. The vesicle forces enter the right-hand sides v and g of the ELS, so that the combined wall-vesicle flow field satisfies the no-slip condition on the channel walls and periodicity across the unit cell, without any double-layer contributions.

Stacking the two block equations yields the final periodic single-layer BIE system:

(26) $$\left[\begin{array}{cc} A & B\\ C & Q \end{array}\right]\left[\begin{array}{c} \tau \\ c \end{array}\right]=\left[\begin{array}{c} v\\ g \end{array}\right].$$

Solving this rectangular system returns both the wall force density τ and the proxy coefficients c, producing a velocity field that is periodic along the channel, satisfies no-slip boundary conditions on the walls, and incorporates the applied pressure driving.

The flow-field parameters used in the simulations are summarized in Table 1.

#### 2.2.2. Material Field

The membrane phase-evolution Equation (3) is numerically stiff because it contains fourth-order spatial derivatives. Consequently, explicit time-marching schemes require prohibitively small time steps. To address this stiffness, the time-marching scheme uses the small-scale decomposition (SSD) approach of [30]; see [12,22] for detailed descriptions of this method applied to vesicle flows.

This decomposition originates from the SSD of the surface mass flux J, for which the stiff small-scale contribution is

(27) $$J∼-\nu a\epsilon \;\psi_{sss}$$

under the local inextensibility condition, which preserves local arclength while allowing the membrane shape to deform. Here *a* is the dimensionless line-tension parameter, ϵ is the interfacial-thickness parameter, and ν is the phase mobility.

Then the governing equation is split to isolate the dominant stiff linear SSD from the smooth non-stiff nonlinear large-scale remainder:

(28) $$\psi_{t}=-\nu a\epsilon \;\psi_{ssss}+N\left(s,t\right),$$

where N(s,t) is the non-stiff nonlinear remainder encapsulating all large-scale effects such as surface advection, lipid-lipid intermolecular interactions, and hydrodynamic coupling to the bulk fluid phase.

Finally, the split equation is mapped to the Fourier wavenumber domain using the Fast Fourier transform, diagonalizing the linear diffusion operator for simplified temporal integration.

(29) $${\hat{\psi }}_{t}\left(k,t\right)=-\nu a\epsilon {\left|k\right|}^{4}\hat{\psi }\left(k,t\right)+\hat{N}\left(k,t\right)$$

where $\hat{\psi }\left(k,t\right)=F\left\{\psi \left(s,t\right)\right\}$ is the Fourier transform of ψ(s,t) and *k* denotes the Fourier wavenumber. Spatial derivatives are converted to algebraic operations: the fourth-order derivative $\psi_{ssss}$ becomes ${\left(ik\right)}^{4}\hat{\psi }={\left|k\right|}^{4}\hat{\psi }$ in wavenumber space. Finally, a second-order exponential time-differencing scheme is used to advance the Fourier-space solution, with the nonlinear remainder evaluated in physical space by the inverse FFT.

The vesicle parameters used in the simulations are summarized in Table 2.

The present simulations use 512 membrane discretization points. This choice is informed by previous two-dimensional wrinkling calculations for homogeneous and multicomponent vesicles, in which 512- and 1024-point results were nearly identical [31]. The wrinkling configurations contained finer spatial variations than the pointed shapes considered here. Previous numerical experience indicates that approximately 128 points can describe the overall bending deformation, whereas the narrower composition-transition regions generally require 256 points or more. We therefore use 512 points for both membrane geometry and the phase field.

## 3. Numerical Results

We first assess the phase-field solver using a benchmark problem. Then we test the vesicle dynamics in the Poiseuille flow.

### 3.1. Evolution of the Phase Field

The phase-field solver is tested by using a benchmark problem in which the vesicle geometry is held fixed and only the membrane composition is allowed to evolve, with the surface flux prescribed by Equation (9). The average phase concentration is set to $\bar{\psi }=0.5$, corresponding to equal fractions of the red and blue phases.

Figure 1 shows the resulting phase-separation process on a highly elongated vesicle with a major-to-minor semi-axis ratio of 1:0.2, as illustrated in Figure 1a. The corresponding nondimensional curvature is shown in Figure 1b. Owing to the elongated geometry, the curvature is strongly localized near the two vesicle tips and reaches a maximum value of approximately H=39.0. Here, α∈[0, 1] denotes the normalized arclength coordinate along the membrane.

The phase-field parameters are set to ν=0.01, a=2000, and ϵ=0.05, and these values are used throughout the subsequent simulations unless otherwise stated. The mobility ν sets the characteristic diffusion timescale, while ϵ controls the spatial extent of the diffuse transition region [12,22]. These parameters were chosen to promote relatively rapid phase reorganization, so that phase separation is largely completed before the vesicle undergoes substantial deformation. The resulting equilibrium phase distribution exhibits three characteristic features. First, the phase field approaches the two pure states, $\max_{\alpha \in [0,\;1]}\psi \left(\alpha \right)\approx 1$ and $\min_{\alpha \in [0,\;1]}\psi \left(\alpha \right)\approx 0$. Second, the diffuse interfaces remain relatively narrow compared with the total membrane length, given $\min|\alpha_{\psi (\alpha )\leq 0.2}-\alpha_{\psi (\alpha )\geq 0.8}|=0.0732$. Third, four distinct phase boundaries form along the membrane, as shown in Figure 1c. The resulting phase distribution reflects the competition between the phase-separation energy and the curvature-dependent bending energy on the highly elongated vesicle.

The normalized interface width of 0.0732 describes a diffuse transition along α∈[0, 1] for the selected parameters; it is not a quantitatively calibrated physiological membrane-interface thickness. For the examined mean compositions within $0.2<\bar{\psi }<0.8$, the stiffer phase preferentially occupies lower-curvature regions, whereas the softer phase accommodates higher curvature. At $\bar{\psi }=0.5$, equal phase fractions on a closed membrane produce a more complex arrangement: domain placement reflects the combined effects of curvature, interface energy, and conserved composition, rather than a strictly pointwise relation between stiffness and curvature.

The fixed-geometry benchmark tests the phase-field component in isolation. For a closed, locally inextensible moving membrane, integration of Equation (3) gives $dM_{\psi }/dt=\oint_{S}J_{s}\;ds=0$, so total phase mass is conserved at the continuum level during deformation. In the coupled simulations below, the evolution of ψ(α,t) for $\bar{\psi }=0.3$, 0.5, and 0.7 also shows the formation of domains with ψ close to 0 and 1 on deforming membranes. Also note that the composition histories use a different color map from the vesicle snapshots to improve visibility; both display the same composition variable. In the shape snapshots, the red phase is softer (ψ≈0) and the blue phase is stiffer (ψ≈1).

### 3.2. A Homogeneous Vesicle in the Channel

We begin by examining the dynamics of homogeneous vesicles in Poiseuille flow within a narrow channel. Two representative values of the bending rigidity are considered: $B=3.0\times 10^{-19}$ J (red) and $B=15.0\times 10^{-19}$ J (blue), corresponding to the reference bending rigidity and five times the reference value, respectively. For each bending rigidity, the simulations are initialized with an elliptical vesicle centered on the channel centerline at three distinct inclination angles, θ(0)=0, π/4, and π/2.

Figure 2 compares the transient evolution and long-time configurations of homogeneous vesicles for the two bending rigidities and three initial orientations. For the reference bending rigidity, the vesicle relaxes to a centerline-symmetric bullet-like shape irrespective of its initial orientation, as shown in Figure 2a. Increasing the bending rigidity leads to changes in both the transient deformation and the long-time configuration. In particular, for $B=15.0\times 10^{-19}\;J$, a horizontally initialized vesicle evolves toward a centerline-symmetric dumbbell-like shape with an enlarged leading end (an unstable equilibrium state), whereas vesicles initialized at θ(0)=π/4 and π/2 relax to bullet-like configurations, as shown in Figure 2b.

### 3.3. A Multicomponent Vesicle in the Channel

Next we investigate the dynamics of a multicomponent vesicle in Poiseuille flow by changing the average phase concentration and initial orientation at a fixed fivefold bending-rigidity contrast. The microcirculation of SCD is characterized by the transport of mechanically heterogeneous RBCs through vessels whose dimensions are comparable to the cell size. In this confined environment, small variations in membrane mechanical properties can strongly influence cell deformation, migration, and flow resistance. In particular, repeated sickling–unsickling cycles associated with hemoglobin S polymerization modify membrane mechanics and generate populations of RBCs with heterogeneous deformability. Motivated by this physiological setting, we next investigate how hydrodynamic confinement regulates the dynamics of multicomponent vesicles with composition-dependent bending rigidity. Unlike homogeneous vesicles, whose mechanical response is determined by a single bending modulus, multicomponent vesicles possess an internal mechanical landscape that evolves through phase separation and couples directly to the surrounding flow.

#### 3.3.1. Vesicle Composed of Two Phases

The vesicle dynamics are highly sensitive to the initial phase distribution. In this section, the initial phase field is prescribed as a nearly uniform distribution with a small perturbation, $\psi \left(\alpha ,0\right)=\bar{\psi }+p\cos\left(4\pi \alpha \right)$, where p=0.001. This perturbation preserves the symmetry of the initial phase distribution while triggering phase separation.

Figure 3 shows the vesicle dynamics for $B_{1}=3.0\times 10^{-19}\;J$, $B_{2}=5B_{1}$, and $\bar{\psi }=0.3$, with three initial inclination angles, θ(0)=0, π/4, and π/2. In all three cases, a similar phase-separation process occurs at early times. The softer red phase preferentially accumulates near the two ends of the initial ellipse, corresponding approximately to α=0 and α=0.5, where the curvature is the largest, while the stiffer blue phase is initially localized in the lower-curvature regions around α=0.25 and α=0.75. As the phase separation proceeds, the two blue domains merge into a single connected domain. The resulting nonuniform distribution of bending rigidity breaks the symmetry of the vesicle and leads to an asymmetric long-time configuration, as shown in Figure 3a–c. Despite their different initial orientations, the three cases approach the same steady asymmetric bullet-like shape, with the stiffer blue phase occupying an extended, relatively flat portion of the leading side. The development of the asymmetry is also reflected in the evolution of the transverse centroid position $y_{c}^{\prime }$ and the inclination angle θ, as shown in Figure 3e,f, respectively. Another notable feature is the transient formation of sharp, high-curvature tips during the deformation induced by the Poiseuille flow, as illustrated in Figure 3a. Such strongly localized curvature is not observed in the corresponding homogeneous-vesicle simulations discussed in Section 3.2.

The initially tilted and vertical vesicles reach this steady configuration at approximately 0.2s. For the initially horizontal vesicle, examination of the evolution beyond 0.4s confirms that the final shape remains unchanged. The superposed final contours in Figure 3d, together with the centroid and angle histories in Figure 3e,f, show convergence to the same configuration. Thus, the initial orientations alter the transient evolution and relaxation time, but not the final shape in these three cases. Approximately 0.4s is the endpoint chosen for displaying the simulation history, not a physical transition or a prescribed equilibration time.

For this minority stiff-phase fraction ($\bar{\psi }=0.3$), the stiffer membrane region favors lower curvature on one side, while the nonuniform curvature distribution can produce a more elongated, pointed configuration with a more pronounced sickle-like asymmetry. Both homogeneous and heterogeneous vesicles can develop broadly bullet-like shapes under confinement. Their similar overall outlines do not imply identical mechanical behavior: heterogeneity modifies the local curvature distribution and the degree of asymmetry.

Figure 4 shows the vesicle dynamics for $B_{1}=3.0\times 10^{-19}\;J$, $B_{2}=5B_{1}$, and $\bar{\psi }=0.5$, with three initial inclination angles, θ(0)=0, π/4, and π/2. Transient sharp tips develop in all three cases, as shown in Figure 4a–c. For θ(0)=0, the vesicle evolves toward a nearly horizontal, asymmetric teardrop-like configuration with a rounded leading end and a pointed trailing tip, a morphology that is not observed in the homogeneous-vesicle simulations reported in Section 3.2. For θ(0)=π/4 and π/2, the vesicle approaches different bullet-shapes for the long-time configuration, which demonstrate the sensitivity of the dynamics to the initial condition and suggest the existence of multiple long-time dynamical states. Figure 4d shows the values of $y_{c}^{\prime }$ and θ over time for the π/4 case, which undergo pronounced transient variations before approaching nearly stationary long-time values. The phase-field evolution in Figure 4e shows rapid phase separation followed by a continuous drift of the phase distribution along the membrane.

Note that the different configurations reached from the three initial orientations at $\bar{\psi }=0.5$ persist over extended simulation histories, with no observed transition away from their respective long-time states.

Figure 5 shows the corresponding dynamics for $\bar{\psi }=0.7$, with $B_{1}=3.0\times 10^{-19}\;J$ and $B_{2}=5B_{1}$. For θ(0)=0, the vesicle approaches a horizontal teardrop-like long-time configuration with a pronounced trailing tip, which is similar to Figure 4a.

For θ(0)=π/4 and π/2, the vesicles evolve toward similar asymmetric long-time shapes, as shown in Figure 5b,c. The transverse centroid positions for the tilted and initially vertical vesicles exhibit transient lateral migration and subsequently relax toward the same final equilibrium, as shown in Figure 5d. Figure 5e further shows a continuous drift of the phase distribution along the membrane for the θ(0)=π/4 case.

#### 3.3.2. Different Channel Width

We then examine changes in vesicle dynamics by varying the channel width *W* while maintaining the pressure gradient and material parameters fixed.

We characterize confinement by χ=R/W, where R=l/(2π)=3.183μm. The widths W=(5.0,7.5,10.0,15.0)μm correspond to χ≈(0.637,0.424,0.318,0.212). For the cell-free Poiseuille profile, $U_{\max}=\Delta PW^{2}/\left(8\;\mu L\right)$, giving $Ca=\Delta PWR^{3}/\left(4LB_{1}\right)\approx \left(12.9,19.4,25.8,38.8\right)$ under the definition in Section 2.1.2. Thus, varying *W* at fixed pressure gradient changes both confinement and the flow scale.

We first examine the influence of channel width *W* on the dynamics of a homogeneous reference vesicle. Four channel widths, W=5.0, 7.5, 10.0, and 15.0μm, are considered under the same pressure gradient. The initial configuration is an elliptical vesicle with its major axis aligned approximately with the flow direction. A small initial rotation of π/180 is introduced in selected cases to avoid artificial symmetry constraints and allow the system to evolve toward its dynamically selected state. These simulations provide homogeneous reference cases at the same channel widths and pressure gradient for qualitative comparison with the multicomponent vesicles.

For the homogeneous vesicle, decreasing the channel width enhances the interaction between the membrane and the channel walls, thereby restricting transverse deformation and modifying the available shape degrees of freedom. Under strong confinement, the vesicle experiences increased geometric constraints and develops a wall-guided configuration, as shown in Figure 6a–d. As the channel becomes wider, the influence of the walls weakens, allowing larger deformation and recovery toward the characteristic bullet-like states observed in confined vesicle flows, as shown in Figure 6e–h.

We then consider a multicomponent vesicle with average phase concentration $\bar{\psi }=0.3$, bending rigidities $B_{1}=3.0\times 10^{-19}\;J$ and $B_{2}=15.0\times 10^{-19}\;J$, corresponding to a fivefold contrast between the two membrane phases. The results are summarized in Figure 7 for the same range of channel widths. When the channel width is relatively large (W=10.0 and 15.0μm), the vesicle develops an asymmetric bullet-like configuration accompanied by slow tank-treading motion. The asymmetric morphology originates from the coupling between phase distribution and curvature: the softer phase preferentially occupies highly curved regions, whereas the stiffer phase stabilizes flatter membrane regions. Consequently, the vesicle no longer possesses the reflection symmetry observed in homogeneous systems.

As the channel width decreases to W=5.0 and 7.5μm, stronger confinement suppresses large-scale shape rearrangement and changes the mode of membrane motion. The vesicle continues to exhibit slow tank-treading behavior, but the classical bullet-like configuration is replaced by more constrained morphologies. This transition demonstrates that confinement does not simply modify the magnitude of deformation; rather, it changes the balance between hydrodynamic forcing, bending resistance, and phase-dependent mechanical adaptation.

The influence of confinement becomes more pronounced when the average phase concentration is increased to $\bar{\psi }=0.5$. In this case, intermediate confinement produces periodic shape oscillations rather than steady tank-treading motion. For W=7.5 and 10.0μm, the vesicle undergoes a breathing-like state characterized by coupled oscillations of the shape, centroid position, and phase distribution, as shown in Figure 8b,c and Figure 9. The phase field evolves periodically along the membrane, but the phase motion is not uniform in time. The evolution of membrane composition is coupled to deformation: compression and elongation modify the curvature distribution, which affects the rearrangement of the mechanical domains.

We interpret the complex breathing and wagging dynamics at $\bar{\psi }=0.5$ as arising from the coupling between equal fractions of stiff and soft phases under the closed-membrane constraint. Small shifts in the phase distribution modify the local bending rigidity and thereby alter membrane deformation. For the chosen parameters, the relatively strong phase-energy contribution and rapid phase reorganization motivate an approximately quasi-equilibrial interpretation of the response to curvature changes. This is a physical interpretation, rather than a measured phase-lag result. The particularly complex dynamics at equal phase fractions are also consistent with the behavior reported in shear flow [22].

The breathing example in Figure 9 has a period of approximately 0.3s. The corresponding full simulation history extends beyond 1.6s and contains several cycles; Figure 9 displays one representative cycle.

The coupled deformation–composition dynamics illustrates how mechanically heterogeneous membranes respond to confined flow. The homogeneous reference calculations provide a qualitative comparison with the multicomponent cases. Additional calculations with uniform dimensionless bending rigidities between 0.2 and 10 approached time-independent configurations without pronounced asymmetry, particularly in wider channels. Within these comparisons, a uniform bending modulus did not reproduce the observed combination of asymmetric shapes and time-dependent composition–shape coupling. This observation concerns the tested cases rather than a quantitative exclusion of every possible fitted effective modulus.

For the widest channel considered, W=15.0μm, the vesicle exhibits a different oscillatory regime. Instead of breathing, the vesicle undergoes wagging motion, characterized by periodic changes in orientation and lateral displacement, as shown in Figure 10. During one wagging cycle, the phase distribution remains coupled to the changing membrane geometry. This behavior highlights the effect of channel geometry on the observed dynamics: changing the width modifies both confinement and the Poiseuille flow scale while keeping the membrane properties unchanged.

Finally, simulations with $\bar{\psi }=0.7$ show trends qualitatively similar to those obtained for $\bar{\psi }=0.3$. The vesicles remain sensitive to confinement, with narrow channels promoting constrained tank-treading states and wider channels allowing more complex deformation dynamics. These results indicate that the transition between dynamical modes is controlled not by phase fraction alone, but by the combined effects of phase composition, bending-rigidity contrast, and hydrodynamic confinement, as shown in Figure 11.

Overall, the channel-width study demonstrates that membrane heterogeneity modifies the curvature distribution, shape asymmetry, and coupled phase dynamics of vesicles in confined flows. In contrast to homogeneous vesicles, where confinement primarily selects among shape families, multicomponent vesicles exhibit an additional layer of mechanical regulation arising from the redistribution of membrane phases. This mechanism provides a qualitative framework for studying mechanically heterogeneous membranes under confinement; the consequences for RBC transport require additional physiological modeling and transport measurements.

Here tank-treading, breathing, and wagging describe the observed modes of motion. Tank-treading at $\bar{\psi }=0.3$ and 0.7 in wider channels is comparatively persistent, whereas $\bar{\psi }=0.5$ exhibits more complex nonlinear dynamics. For the states classified as steady, the shape and the monitored composition behavior show no appreciable long-time change, and the corresponding dimensionless velocity changes or material fluxes are below $10^{-6}$. This refers to the steady observables and does not imply that membrane material points cease tank-treading. The persistent states are interpreted as locally sustained configurations in the highly symmetric deterministic model, rather than as a perturbation-based stability classification.

## 4. Discussion

The present study establishes a coupled phase-field and boundary-integral framework for describing multicomponent vesicles in confined Poiseuille flow. By incorporating composition-dependent bending rigidity, the model reveals that membrane heterogeneity qualitatively modifies vesicle dynamics compared with the homogeneous reference cases. Although both homogeneous and heterogeneous vesicles can develop broadly bullet-like shapes under confinement, the heterogeneous membrane exhibits symmetry breaking, composition-correlated curvature localization, and coupled phase redistribution. These effects lead to asymmetric configurations and, depending on the degree of confinement, breathing and wagging motions.

The main contribution of this work is the identification of coupled interactions among membrane composition, curvature, and hydrodynamics as a mechanism controlling vesicle morphology and motion in narrow channels. These findings extend classical homogeneous-vesicle theories and provide a mechanistic explanation for how membrane heterogeneity may influence transport and deformation in microvascular environments.

The relevance to RBCs and SCD is qualitative: the model isolates coupling among membrane heterogeneity, curvature, and confined flow. The present calculations do not quantify hydraulic resistance, cell trapping, or vaso-occlusion. The possibility that multiple cells with narrow, high-curvature tips could pack together and obstruct a vessel is a hypothesis for future study, not a conclusion established by these single-vesicle simulations.

Microfluidic and computational studies provide evidence for a qualitative connection between altered RBC mechanics and impaired microvascular transport. Du et al. [32] observed hypoxia-induced sickle-cell trapping and quantified transit velocities and obstruction ratios. Combining experiments with simulations, Li et al. [33] showed that cell shape, orientation, and deformability influence passage and blockage at capillary-sized constrictions. Higgins et al. [34] demonstrated collective jamming upon deoxygenation and restoration of flow upon reoxygenation. Together, these studies support the biological motivation for relating abnormal cell mechanics and morphology to impaired transport. However, they do not establish that geometric asymmetry or localized high curvature per se is sufficient to increase hydrodynamic resistance or promote cell trapping. Therefore, the direct relationship between the high-curvature morphologies observed here and increased flow resistance, cell trapping, or vaso-occlusion remains to be established through dedicated transport measurements and more physiologically realistic simulations incorporating cell–cell interactions, adhesion, and realistic vessel geometry.

Several limitations remain. The current model assumes equal internal and external viscosities, two-dimensional geometry, locally inextensible membranes, and a linear interpolation of bending rigidity. These assumptions isolate the role of phase-dependent bending mechanics but limit quantitative comparison with biological cells. Future work should incorporate viscosity contrast, three-dimensional geometries, cytoskeletal elasticity, membrane tension regulation, and realistic microvascular networks.

The fivefold rigidity contrast was chosen as a compromise. A smaller contrast, such as $B_{2}/B_{1}=2$, produced less pronounced morphological differences, whereas similar behavior was observed for ratios between 4 and 6. At $B_{2}/B_{1}=10$, the calculations could become numerically unstable and produce negative values of ψ, which are not accepted as physically admissible phase concentrations. This numerical limitation does not imply that a larger physical bending contrast is impossible. The sensitivity to nonlinear interpolation laws has not been tested here.

The coupled dynamics is strongly nonlinear, especially at equal phase fractions. Similar complexity has been observed in shear-flow dynamics and in wrinkling under time-dependent extensional flow [22,31]. The persistent configurations here occur in a smooth, highly symmetric channel without thermal fluctuations. Wall irregularities, changes in the imposed flow, or thermal fluctuations may disrupt these locally sustained states, so their reproducibility in experiments cannot be inferred from persistence in the idealized simulations alone. The earlier wrinkling study [31] also provides a comparison of local deformation scales in homogeneous and multicomponent membranes; the present channel study focuses on how heterogeneous bending can promote asymmetric, sickle-like configurations.

At the bilayer scale, curvature-dependent leaflet packing and interleaflet stresses may provide additional mechanical contributions that are not represented by the present single-surface B=B(ψ) description. Recent experimental observations by Wang et al. [35] suggest that membrane curvature can alter thermodynamic properties associated with collective lipid rearrangements; incorporating such effects would require leaflet-resolved variables and additional constitutive relations for packing and interleaflet mechanics. Composition-dependent spontaneous curvature may introduce an additional mechanism for phase sorting. In that extension, both the preferred curvature and the bending modulus would contribute to the chemical potential; assessing their effects on the highly curved tips would require solving the extended model. A dynamically evolving preferred curvature could also be considered in future work to represent changes associated with sustained curvature, affecting both bending energy and bending force. 

## Figures and Tables

**Figure 1:**
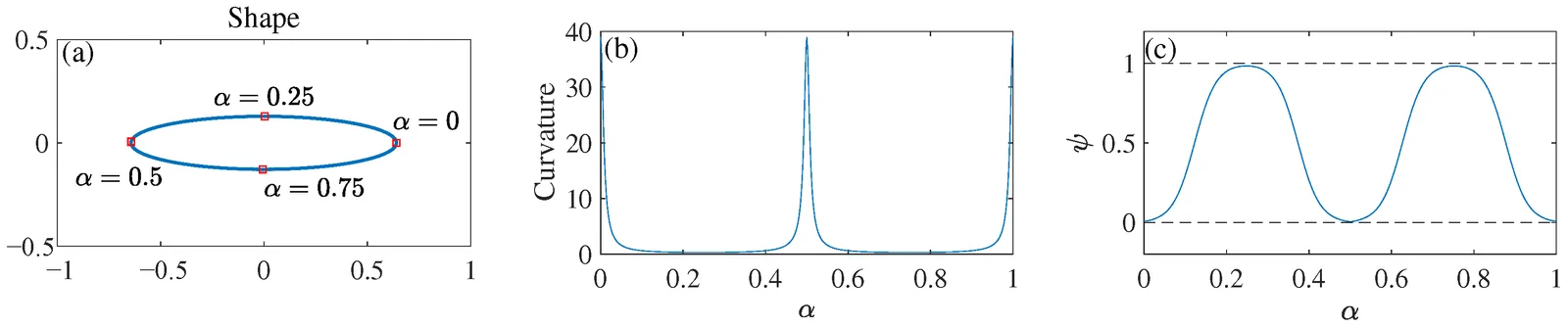
(**a**) Shape of the vesicle. (**b**) Nondimensional curvature of the vesicle boundary. (**c**) Phase field ψ after phase separation.

**Figure 2:**
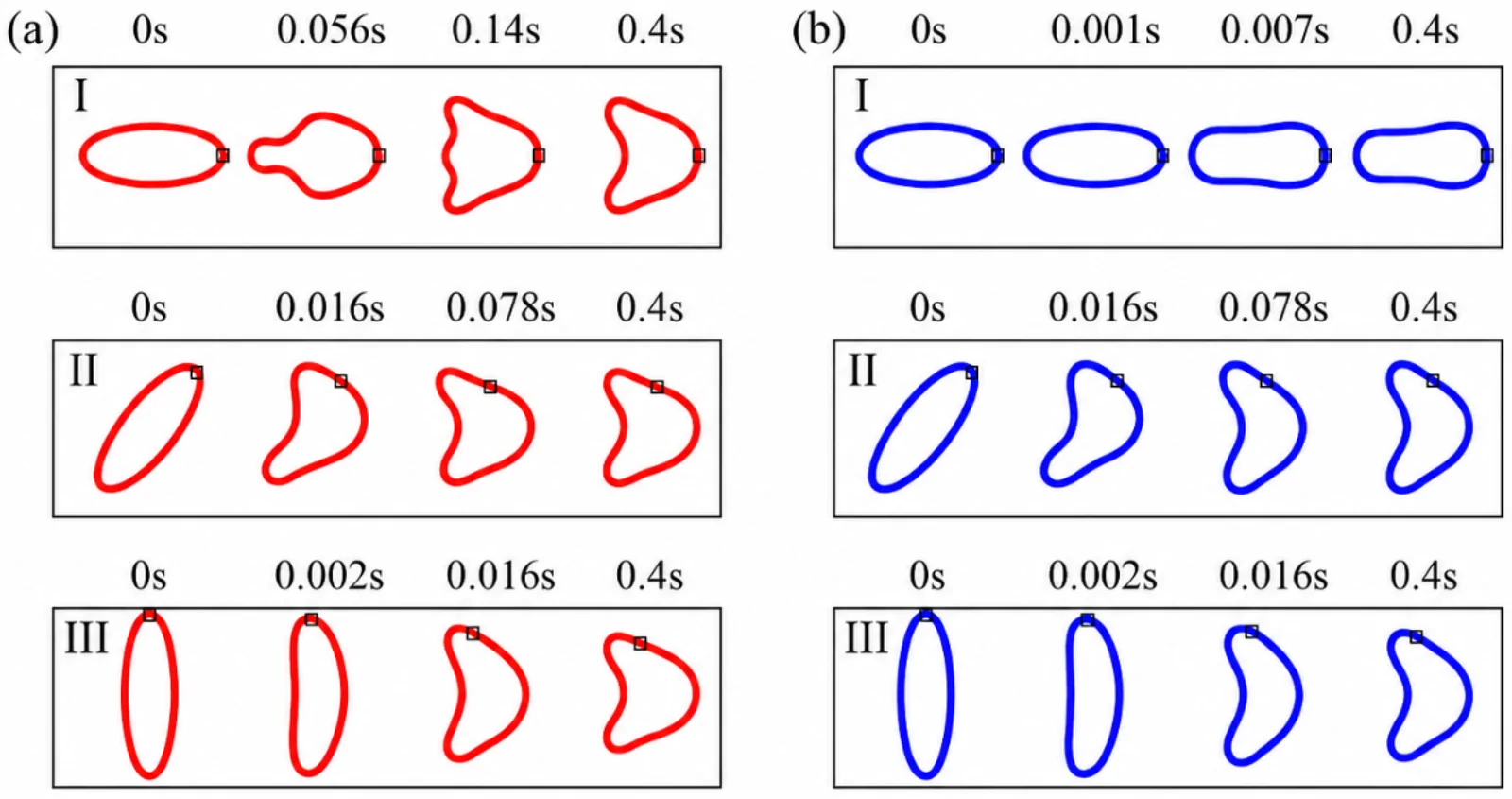
Dynamics of a homogeneous vesicle in Poiseuille flow. (**a**) Reference bending rigidity $B=3.0\times 10^{-19}\;J$ for three initial inclination angles: (I) θ(0)=0, (II) θ(0)=π/4, and (III) θ(0)=π/2. (**b**) Higher bending rigidity $B=15.0\times 10^{-19}\;J$ for the same initial inclination angles.

**Figure 3:**
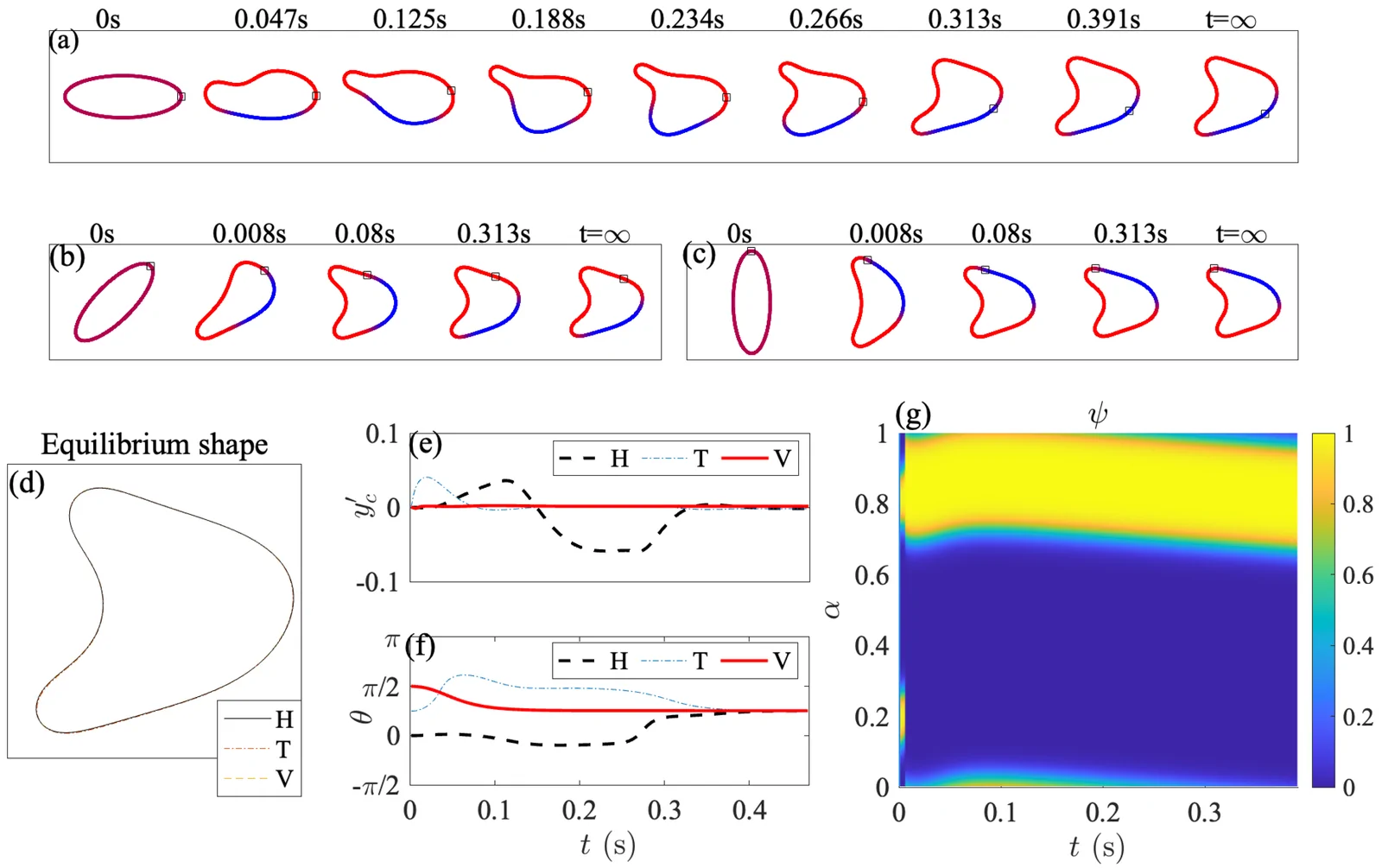
Vesicle dynamics for $B_{1}=3.0\times 10^{-19}\;J$, $B_{2}=5B_{1}$, and $\bar{\psi }=0.3$: (**a**) θ(0)=0; (**b**) θ(0)=π/4; (**c**) θ(0)=π/2; (**d**) superposed steady-state contours for the three initial orientations; (**e**) evolution of the transverse centroid position $y_{c}^{\prime }$; (**f**) evolution of the inclination angle θ; (**g**) spatiotemporal evolution of ψ(α,t) for θ(0)=π/2. The labels *H*, *T*, and *V* denote the horizontal [θ(0)=0], tilted [θ(0)=π/4], and vertical [θ(0)=π/2] initial orientations, respectively.

**Figure 4:**
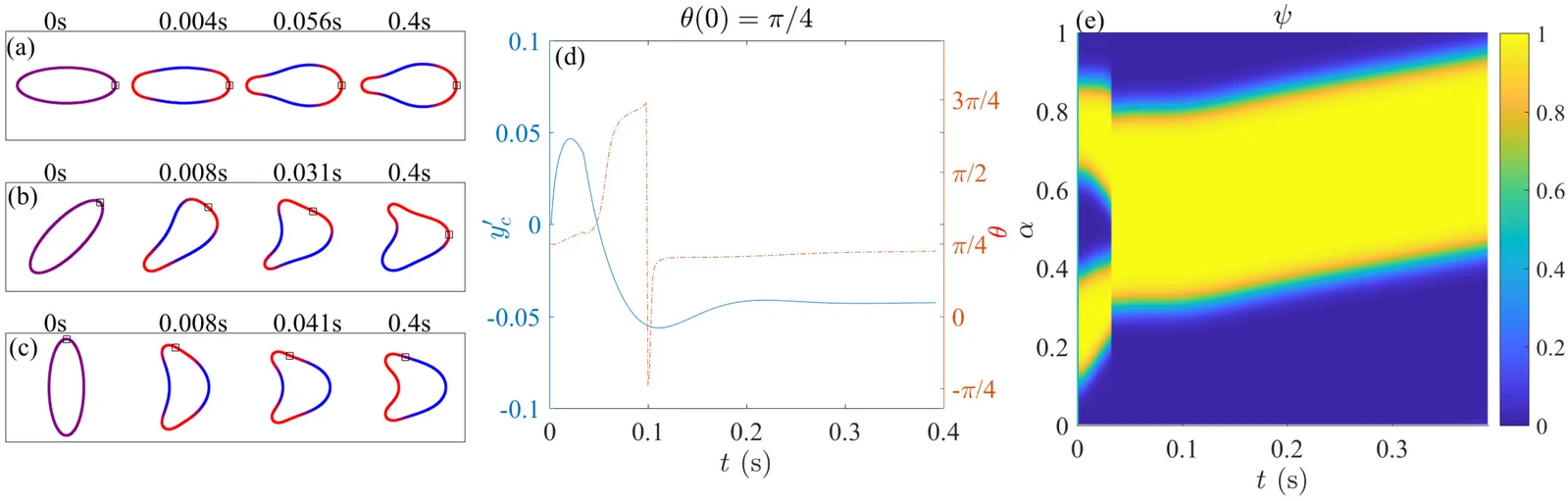
Vesicle dynamics when $B_{1}=3.0\times 10^{-19}\;J$, $B_{2}=5B_{1}$, and $\bar{\psi }=0.5$: (**a**) θ(0)=0; (**b**) θ(0)=π/4; (**c**) θ(0)=π/2; (**d**) evolution of $y_{c}^{\prime }$ and θ for case θ(0)=π/4; (**e**) ψ(α,t) for case θ(0)=π/4.

**Figure 5:**
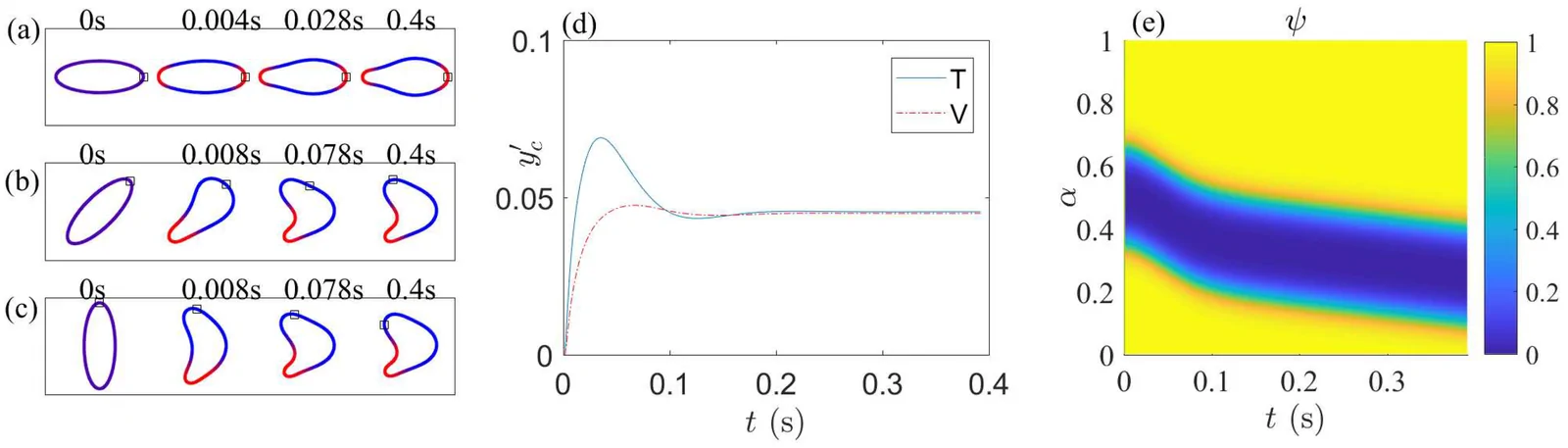
Vesicle dynamics when $B_{1}=3.0\times 10^{-19}\;J$, $B_{2}=5B_{1}$, and $\bar{\psi }=0.7$: (**a**) θ(0)=0; (**b**) θ(0)=π/4; (**c**) θ(0)=π/2; (**d**) evolution of $y_{c}^{\prime }$; (**e**) ψ(α,t) for case θ(0)=π/4.

**Figure 6:**
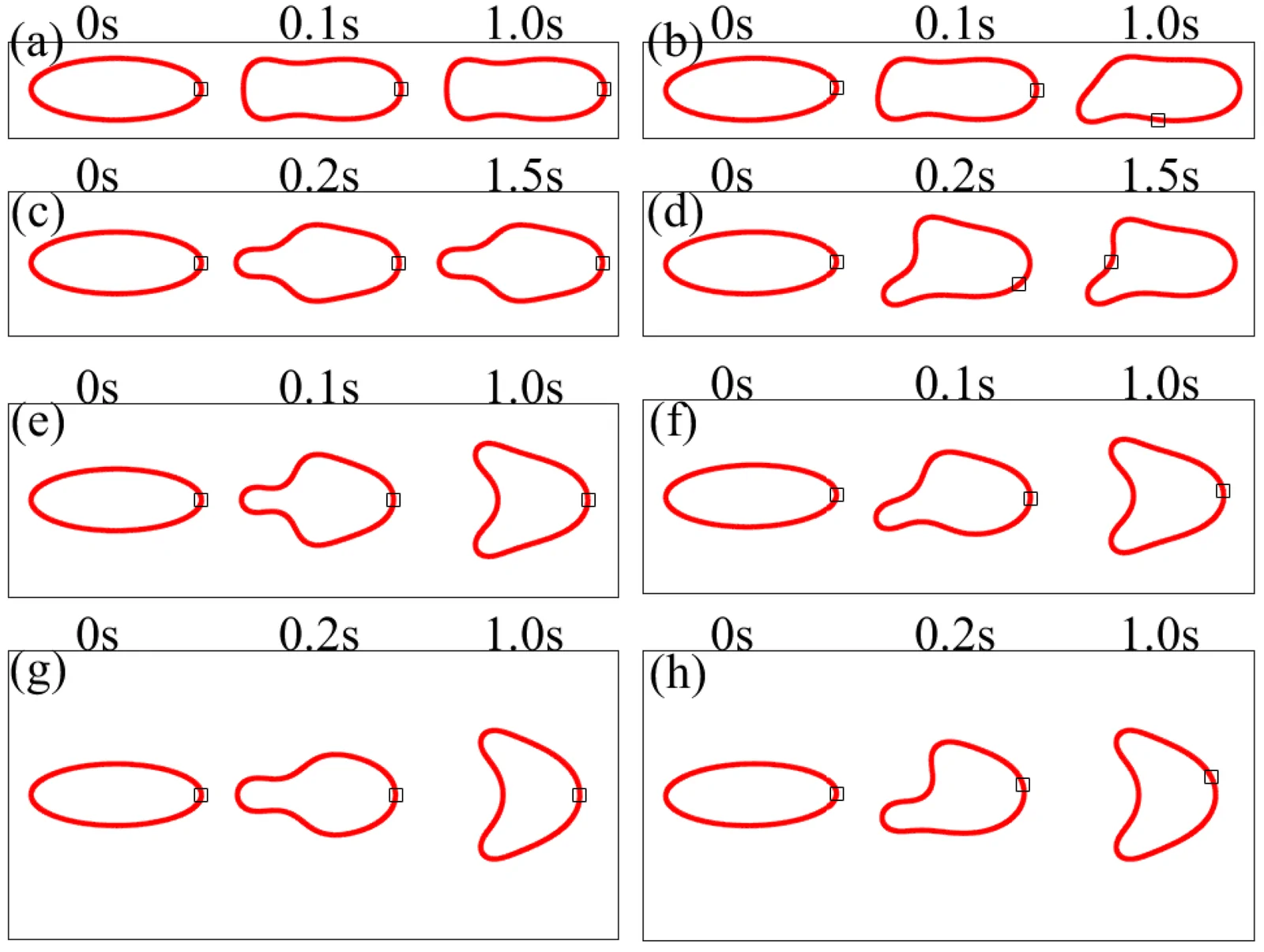
Homogeneous-vesicle dynamics when $B_{1}=3.0\times 10^{-19}\;J$: (**a**) W=5.0μm with an initially horizontal orientation, marked as (H); (**b**) W=5.0μm with the initial condition slightly tilted (π/180 counterclockwise), marked as (T); (**c**) W=7.5μm (H); (**d**) W=7.5μm (T); (**e**) W=10.0μm (H); (**f**) W=10.0μm (T); (**g**) W=15.0μm (H); (**h**) W=15.0μm (T).

**Figure 7:**
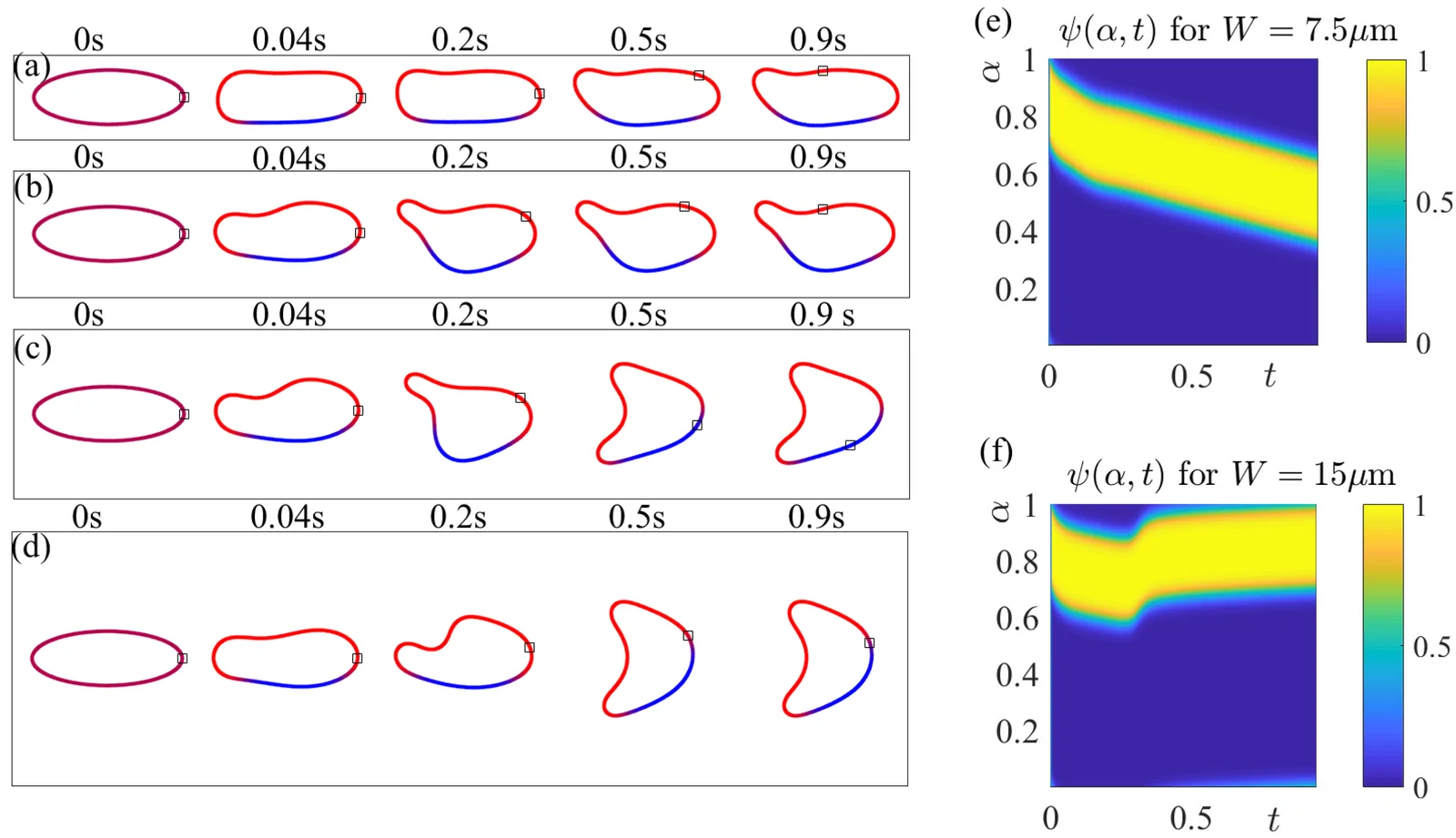
Vesicle dynamics when $B_{1}=3.0\times 10^{-19}\;J$, $B_{2}=15.0\times 10^{-19}\;J$, and $\bar{\psi }=0.3$: (**a**) W=5.0μm; (**b**) W=7.5μm; (**c**) W=10.0μm; (**d**) W=15.0μm with an initially horizontal orientation; (**e**) ψ(α,t) for W=7.5μm; (**f**) ψ(α,t) for W=15.0μm.

**Figure 8:**
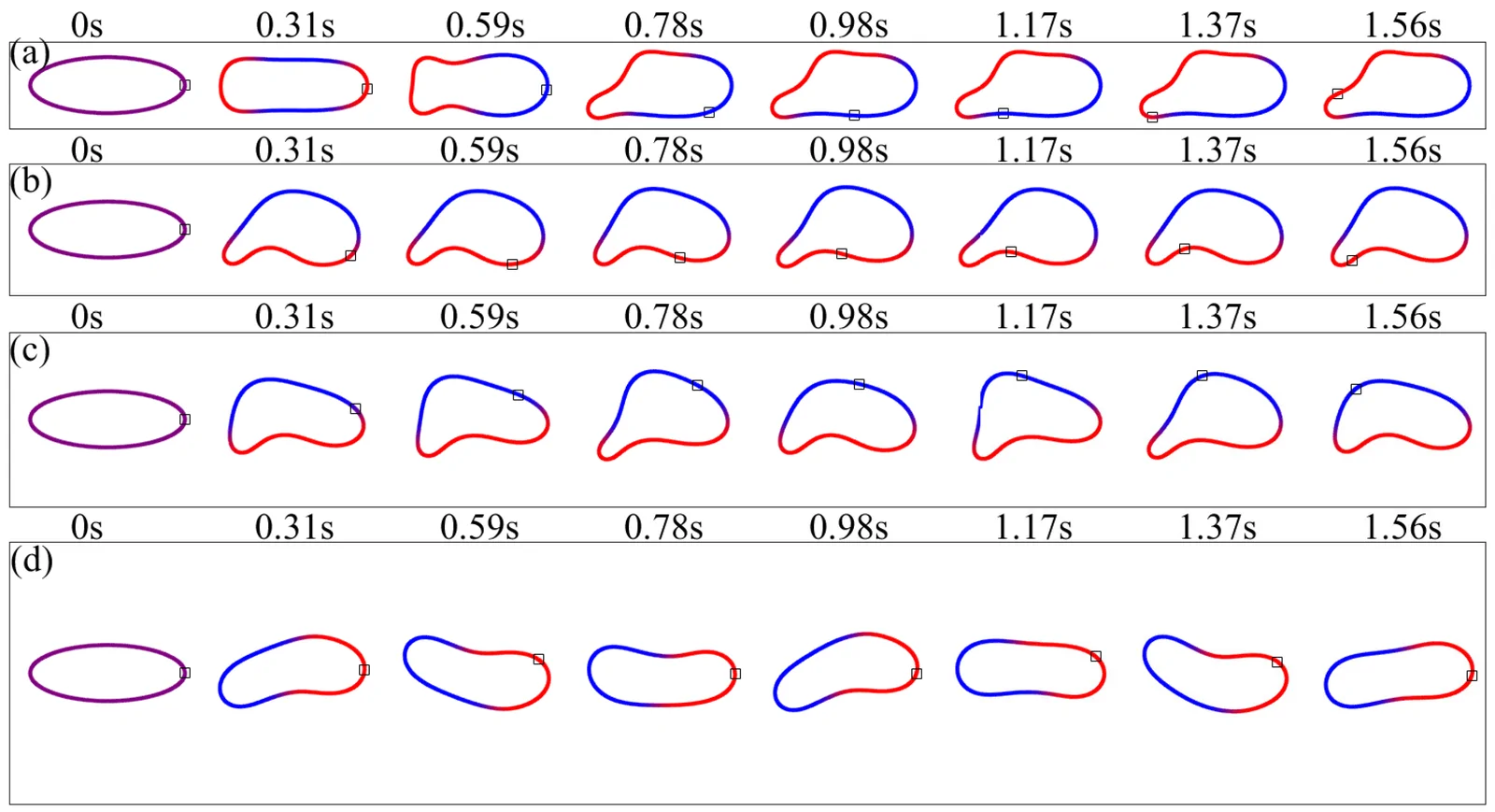
Vesicle dynamics when $B_{1}=3.0\times 10^{-19}\;J$, $B_{2}=15.0\times 10^{-19}\;J$, and $\bar{\psi }=0.5$: (**a**) W=5.0μm; (**b**) W=7.5μm; (**c**) W=10.0μm; (**d**) W=15.0μm with an initially horizontal orientation.

**Figure 9:**
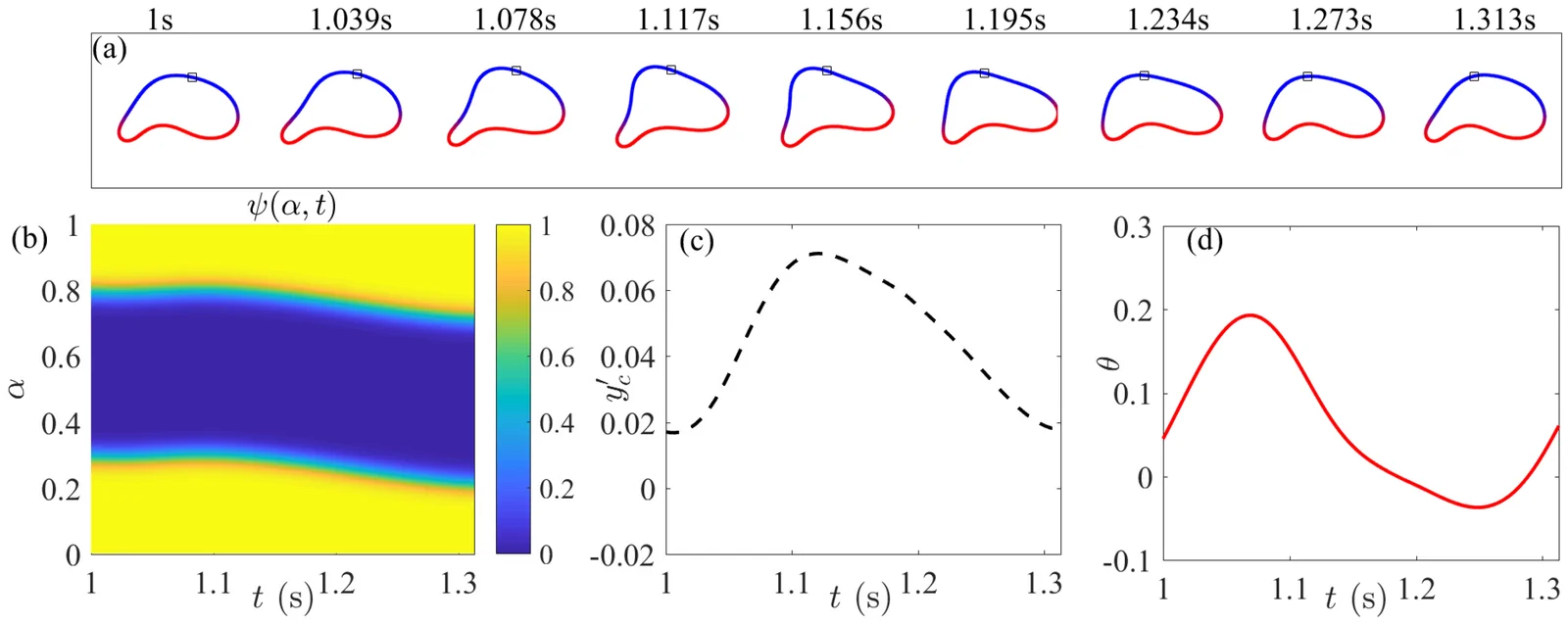
One breathing cycle of the vesicle for W=10.0μm from t=1.000 to 1.313s: (**a**) vesicle shape; (**b**) ψ(α,t); (**c**) $y_{c}^{\prime }\left(t\right)$; (**d**) θ(t).

**Figure 10:**
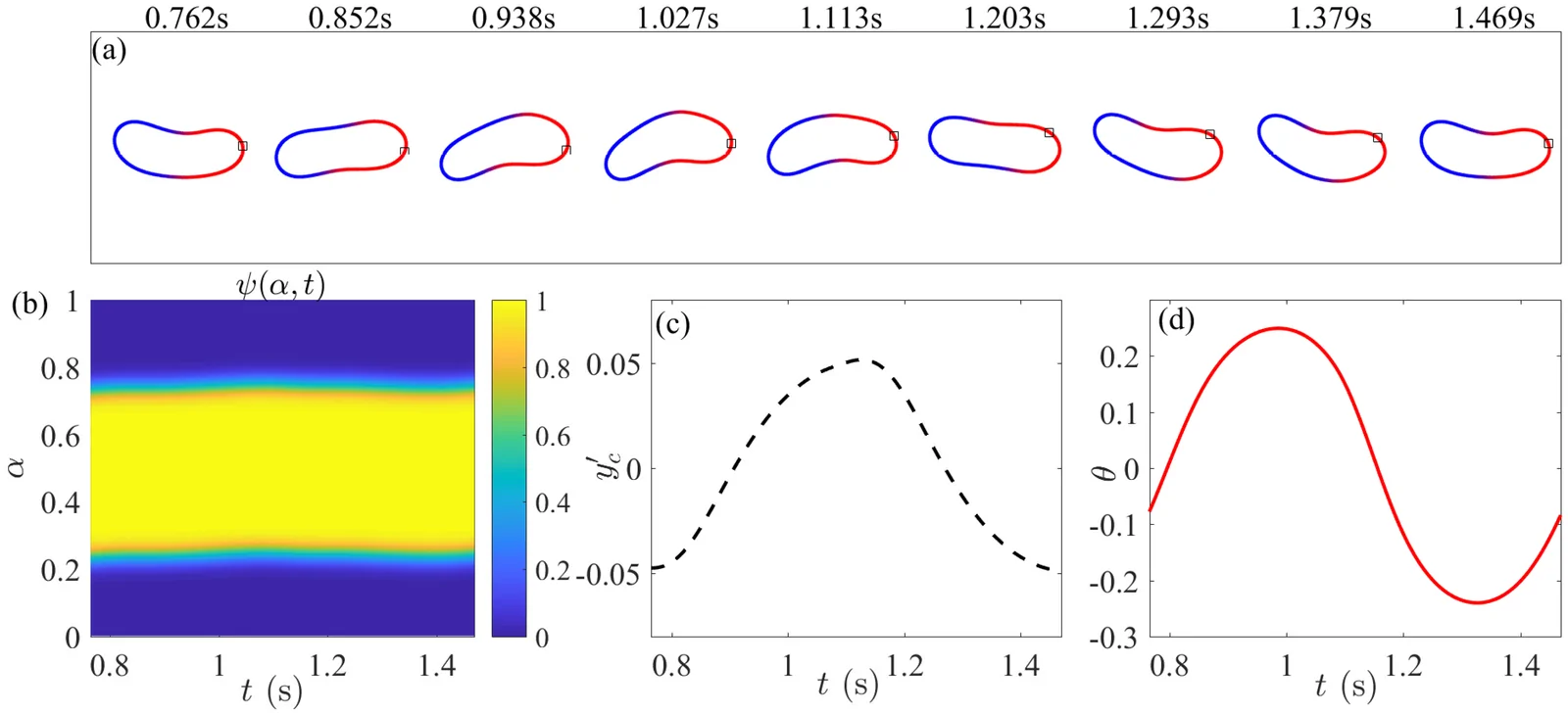
One wagging cycle of the vesicle for W=15.0μm from t=0.762 to 1.469s: (**a**) vesicle shape; (**b**) ψ(α,t); (**c**) $y_{c}^{\prime }\left(t\right)$; (**d**) θ(t).

**Figure 11:**
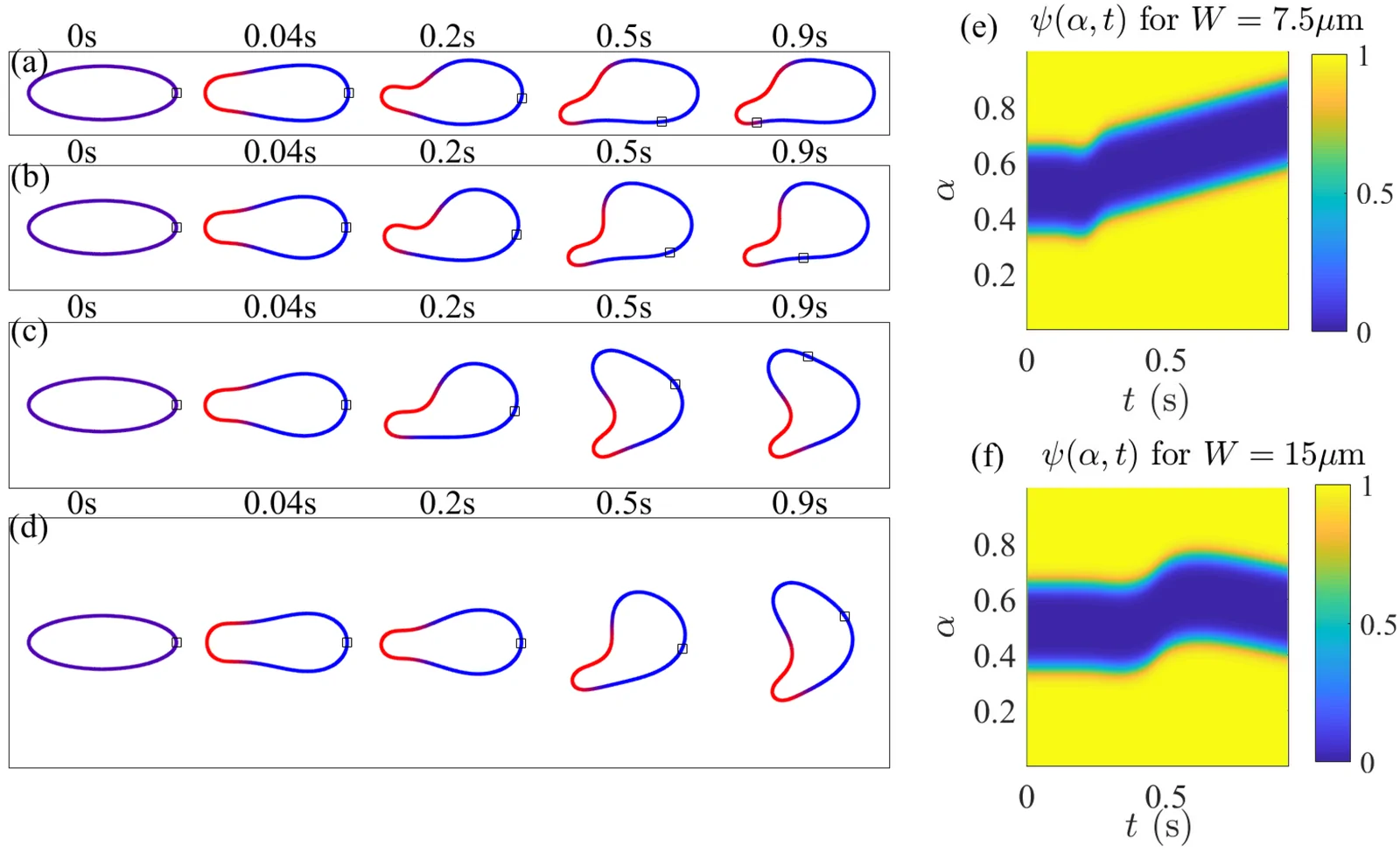
Vesicle dynamics when $B_{1}=3.0\times 10^{-19}\;J$, $B_{2}=15.0\times 10^{-19}\;J$, and $\bar{\psi }=0.7$: (**a**) W=5.0μm; (**b**) W=7.5μm; (**c**) W=10.0μm; (**d**) W=15.0μm with an initially horizontal orientation; (**e**) ψ(α,t) for W=7.5μm; (**f**) ψ(α,t) for W=15.0μm.

**Table 1:** Parameters for the flow field.

Parameter	Notation	Value
Width (μm)	*W*	10
Period (μm)	*L*	44.2
Viscosity (Pa·s)	μ	$1.2\times 10^{-3}$
Pressure drop (Pa)	ΔP	4.25
Mass density (kg/m^3^)	ρ	$10^{3}$
Reynolds number	Re	≈$8.3\times 10^{-3}$

**Table 2:** Parameters for the vesicle.

Parameter	Notation	Value
aspect ratio	AR	1:0.3615
Arclength (μm)	*l*	20
Reference bending rigidity (J)	$B_{1}$	$3\times 10^{-19}$
Magnitude of Phase Energy	*a*	2000
Phase Boundary Gradient	ϵ	0.05
Phase Mobility	ν	0.01

* Note that the phase-field parameters ϵ, *a*, and ν are nondimensional.

## Data Availability

The data supporting the findings of this study are available from the corresponding author upon reasonable request.
